# Elastic Deformation of Soft Tissue-Mimicking Materials Using a Single Microbubble and Acoustic Radiation Force

**DOI:** 10.1016/j.ultrasmedbio.2020.08.012

**Published:** 2020-12

**Authors:** James H. Bezer, Hasan Koruk, Christopher J. Rowlands, James J. Choi

**Affiliations:** ⁎ Department of Bioengineering, Imperial College London, London, United Kingdom; † Mechanical Engineering Department, MEF University, Istanbul, Turkey

**Keywords:** Acoustic radiation force, Bjerknes force, Ultrasound contrast agents, Microbubbles, Cavitation, Drug delivery

## Abstract

Mechanical effects of microbubbles on tissues are central to many emerging ultrasound applications. Here, we investigated the acoustic radiation force a microbubble exerts on tissue at clinically relevant therapeutic ultrasound parameters. Individual microbubbles administered into a wall-less hydrogel channel (diameter: 25–100 µm, Young's modulus: 2–8.7 kPa) were exposed to an acoustic pulse (centre frequency: 1 MHz, pulse length: 10 ms, peak-rarefactional pressures: 0.6–1.0 MPa). Using high-speed microscopy, each microbubble was tracked as it pushed against the hydrogel wall. We found that a single microbubble can transiently deform a soft tissue-mimicking material by several micrometres, producing tissue loading–unloading curves that were similar to those produced using other indentation-based methods. Indentation depths were linked to gel stiffness. Using a mathematical model fitted to the deformation curves, we estimated the radiation force on each bubble (typically tens of nanonewtons) and the viscosity of the gels. These results provide insight into the forces exerted on tissues during ultrasound therapy and indicate a potential source of bio-effects.

## Introduction

Microbubbles are increasingly used as a contrast agent in ultrasound imaging and as a therapeutic agent in ultrasound therapy. The most commonly used microbubbles are composed of a heavy gas encased in a lipid shell. They have a typical diameter of 1–10 µm, which is small enough for them to pass freely through the smallest blood vessels, but large enough that they remain confined within the vasculature.

The most common clinical use of microbubbles is in ultrasound imaging (Cosgrove 2006). They are especially valuable for their ability to image blood flow, and have consequently found widespread applications in cardiovascular medicine (Mulvagh et al. 2008) and in imaging masses in abdominal organs such as the liver (Wilson and Burns 2010). Imaging applications continue to expand and include super-resolution to identify single microbubbles at micrometre-scale spatial resolution (Christensen-Jeffries et al. 2015; Errico et al. 2015) and molecular imaging, whereby ligand-coated microbubbles bind to receptors expressed on vascular endothelial cells (Deshpande et al. 2010; Abou-Elkacem et al. 2015). In these applications, microbubbles increase the returned ultrasound signal by oscillating in response to the imaging pulse. The increased signal provides contrast to the surrounding tissue, thereby enabling the many imaging applications described.

Microbubbles also have a wide range of potential therapeutic applications, including blood–brain barrier permeabilisation (Hynynen et al. 2001; Burgess et al. 2015), thrombolysis (Mathias et al. 2019) and delivery of drugs through cell membranes (van Wamel et al. 2006; Helfield et al. 2016). The therapeutic effects produced are believed to be related to the mechanical forces that the microbubbles exert on vascular endothelial cells, blood vessels and surrounding tissues (Chen et al. 2014; Burgess et al. 2015). However, the exact nature of the forces microbubbles exert on soft tissues during therapy and how they lead to therapeutic effects remain less understood (Roovers et al. 2019).

Research on bubble physics in imaging and therapy has focussed primarily on the volumetric oscillations of microbubbles. The non-linear radial oscillations of bubbles are key to generating significant and distinctive patterns of backscatter in imaging (Faez et al. 2013), and are predicted to exert oscillatory forces on surrounding tissues (Hosseinkhah et al. 2013). Theoretical studies have investigated the forces exerted *via* volumetric oscillations of microbubbles (Hosseinkhah and Hynynen 2012; Wang et al. 2013), which have been found experimentally to deform the walls of blood vessels when exposed to high-amplitude short pulses (Chen et al. 2011, 2012).

However, microbubbles exposed to ultrasound also experience a translational force, typically in the direction of sound propagation, known as the primary acoustic radiation force (sometimes referred to as the primary Bjerknes force) (Leighton 1994; Dayton et al. 2002). The primary radiation force on microbubbles has previously received theoretical and experimental attention for its ability to displace bubbles in a free fluid (Dayton et al. 2002; Blue et al. 2018) and to bring bubbles toward a boundary, as a way of enhancing the contrast of molecular imaging with ligand-targeted microbubbles (Shortencarier et al. 2004; Lum et al. 2006; Frinking et al. 2012).

Less experimental attention has previously been given to the effects that contrast agent microbubbles, at interfaces, driven by acoustic radiation forces may have on tissue. Radiation forces caused by 1-MHz ultrasound have been reported to cause individual microbubbles to tunnel into fibrin clots (Acconcia et al. 2013) and agarose at pressures >1.2 MPa (Caskey et al. 2009). By use of large, concentrated clouds of microbubbles, the primary radiation force has been found to displace blood clots (Wright et al. 2012).

The radiation force bubbles can exert on their surroundings has been proposed as a method of estimating material mechanical properties. Changes in tissue mechanical properties are associated with many pathologies. The radiation force on a single laser-induced bubble embedded in a medium has been investigated as a way to measure the stiffness of tissues, including soft gels and the eye (Erpelding et al. 2005; Ilinskii et al. 2006; Yoon et al. 2011; Shirota and Ando 2015). Large clouds of contrast agent microbubbles have also been reported to reversibly deform soft gels, enabling values related to material stiffness to be extracted (Koruk et al. 2015; Saharkhiz et al. 2018).

Here, the dynamic responses of individual microbubbles at soft gel interfaces exposed to the primary acoustic radiation force are investigated using ultrasound parameters that are typical in therapeutic applications. This is achieved by tracking, with high-speed optical microscopy (frame rate of 4,858 or 31,197 fps), the elastic indentation of a bubble into a soft hydrogel when exposed to ultrasound. The dynamic responses of individual bubbles at three different hydrogel interfaces, with Young's moduli of 2, 4.5 and 8.7 kPa—bulk properties similar to those of soft tissues such as the brain (Kaster et al. 2011; Macé et al. 2011; McKee et al. 2011)—are investigated. In addition to the experimental investigation, a mathematical model was used to extract the radiation force on the bubble and the gel viscosity based on experimental results. The ultrasound parameters used here (1-MHz centre frequency, 10-ms pulse length, peak rarefactional pressures of 0.6–1 MPa) are very similar to those used in applications such as blood–brain barrier permeabilization, both in animals (McDannold et al. 2005; Choi et al. 2011) and in humans (Carpentier et al. 2016; Idbaih et al. 2019). They are also comparable to parameters used clinically in sonothrombolysis (Leeman et al. 2012; De Saint Victor et al. 2014).

## Methods

Individual microbubbles were introduced to wall-less channels in soft hydrogels. When exposed to ultrasound, their motion into the gel was tracked using high-speed microscopy. Their maximum indentation depth and the shape of the indentation curve were used to infer properties of the gel, and the force exerted by the bubble on the gel, using a mathematical model. The maximum bubble indentation depths into the gel are compared for three gel stiffnesses and two channel diameters, to investigate effects caused by confinement within a small and soft blood vessel.

### Microbubble preparation

Microbubbles were manufactured in-house using a previously described protocol (Koruk et al. 2015; Shamout et al. 2015). The microbubble shell consisted of three lipids (Avanti Polar Lipids Inc., Alabaster, AL, USA) from powder—dipalmitoylphosphatidylcholine (DPPC), dipalmitoylphosphatidic acid (DPPA) and dipalmitolyphosphatidylethanolamine–polyethylene glycol 2000 (DPPE-PEG2000)—which were mixed and diluted with glycerol (5% v/v) and saline (80% v/v). Vial headspace was filled with perfluorobutane and mechanically amalgamated for 45 s (Synergy Electronics, Scottsdale, AZ, USA). Microbubbles were extracted from the vial with a 20G syringe needle and then diluted in 0.9% saline. Microbubbles were diluted such that they were well spaced within the channel, at least 100 µm between bubbles to reduce coupling (Schutt et al. 2014). This was achieved at a concentration of approximately 10^6^ microbubbles/mL. The mean bubble radius was 0.66 ± 0.38 µm (Koruk et al. 2015). However, only the larger portion of bubbles were selected for optical tracking (radius >0.5 µm). This was because smaller bubbles did not respond significantly and larger bubbles were better resolved by the camera.

### Gel channel preparation

Polyacrylamide was chosen for the experiments here because of its high optical and acoustic transparency and tuneable mechanical properties allowing gels to be produced with Young's moduli similar to those of soft tissues. Polyacrylamide gel was formed using powdered acrylamide monomer and powdered N,N′-methylene bisacrylamide as a cross-linker. Gels with three different stiffnesses were produced, with acrylamide:bis ratios of 4:0.1, 5:0.15 and 5:0.3 (percentage by mass in de-ionised water). These ratios produced gels with Young's moduli of 2.01 ± 0.75, 4.47 ± 1.19 and 8.73 ± 0.79 kPa, respectively, according to a published protocol (Tse and Engler 2010). The solution was de-gassed and then mixed at room temperature with 0.1 g/100 mL ammonium persulphate and 100 µL/100 mL tetramethylethylenediamine (TEMED) before being left to set for several minutes. All reagents were obtained from Sigma-Aldrich (Dorset, UK).

**Fig. 1:**
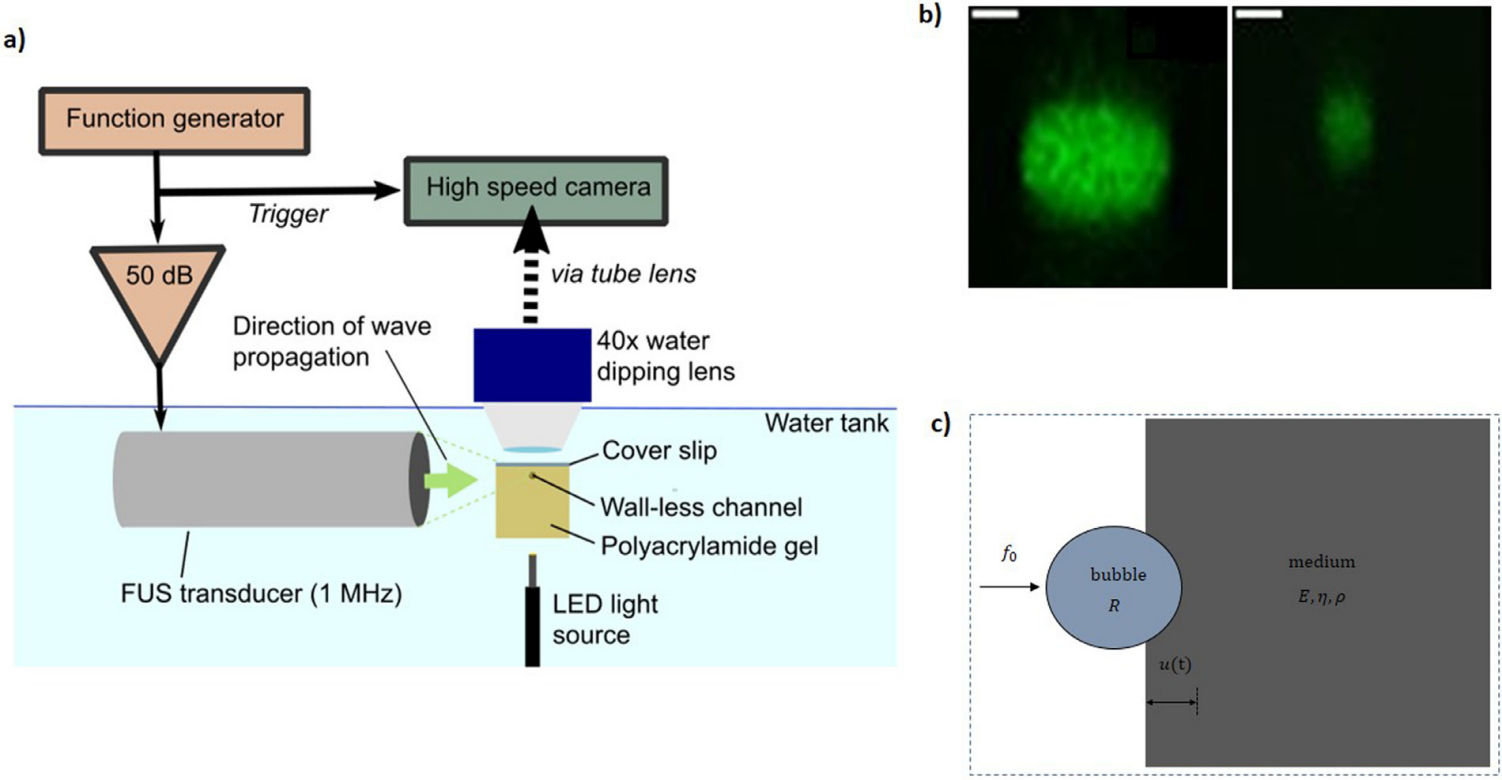
Materials and methods. (a) Experimental setup. A 25- or 100-µm-diameter wall-less channel in soft polyacrylamide gel (Young's modulus: 2–8.7 kPa) was filled with a dilute solution of microbubbles and sonicated at 1 MHz. The motion of the bubble was tracked under a microscope with a high-speed camera. (b) Example cross-sections of wall-less channels in 2-kPa Young's modulus polyacrylamide gels imaged with confocal microscopy after introduction of a solution of quantum dots (left: 100-μm diameter channel, right: 25-μm diameter channel, scale bar = 25 μm). (c) Schematic of mathematical model illustrating a bubble impacting a viscoelastic medium.

Gels were formed in a 3-D-printed U-shaped box with a thin plastic coverslip on top. The volume of the gel was 1.5 cm (width, perpendicular to direction of ultrasound propagation) × 1.5 cm (height) × 1 cm (axial depth) (Fig. 1a). Two opposite ends of the gel remained open, allowing a free acoustic path along the axial direction to minimise reflections. A coverslip was also fixed over a hole in the bottom of the box, allowing the channel to be illuminated from below.

The channel was created using a 25- or 100-μm-diameter nickel/chromium (80:20) wire. The box contained two 30G hypodermic needles, placed facing each other approximately 1 mm from the lower surface of the coverslip, to ensure the channel could be placed close enough to the lens to be imaged clearly. Thirty-gauge needles were chosen, as a smaller diameter enabled bubbles to be more easily introduced into the narrow 25-µm channel. The wire was passed through these needles, and once the wire was in place, the polyacrylamide solution was poured into the box and left to set. The wire was then removed, and a dilute solution of microbubbles was introduced into the channel through the needles using a syringe pump (PHD Ultra, Harvard Apparatus, Holliston, MA, USA) at a flow rate of 0.1 mL/min. No flow was applied during the optical experiments.

### Gel channel characterisation

Mechanical properties of hydrogels can be measured using nano-indentation, atomic force microscopy (AFM) or oscillatory rheometry (Oyen 2014). The gels were made from a published protocol based on the relative concentrations of acrylamide to bisacrylamide (Tse and Engler 2010). The authors of the protocol tested the Young's modulus of polyacrylamide gels with atomic force microscopy, and the values published in this protocol are used here. It should be noted that a wide variation in elasticity (>20%) was reported between identically produced gels and different regions of the same gel.

Polyacrylamide is a hydrogel, and properties of polymers and hydrogels vary at very high frequencies (Smyth et al. 2001). The relevance of low-frequency indentation measurements to high-frequency deformations produced by bubble displacement and radial oscillations (kHz to MHz) is therefore unclear. A mismatch between viscosity estimated from kilohertz-frequency bubble dynamics and low-frequency oscillatory rheometry has previously been observed (Jamburidze et al. 2017).

The acoustic properties of the gel were tested to ensure that the pressure within the gel channel was approximately the same as measured by the hydrophone when the gel was not present. Attenuation spectra of the gels were produced, which confirmed there was minimal scattering and attenuation compared with water. This required use of a 2.25-MHz immersion transducer (Olympus Industrial, Southend-on-Sea, Essex, UK) sending a short broadband pulse (DPR300 pulser/receiver, JSR Ultrasonics, Pittsford, NY, USA) through a 2-cm-thick sample of gel, within a 3-D-printed chamber, to an aluminium reflector (Browne et al. 2003). The reflected pulse was detected by the same transducer, and the frequency spectrum was compared with that received for a pulse transiting the chamber when filled with water. This was performed for each gel stiffness used. There was negligible attenuation by any of the three gels (<<0.5 dB/cm) at 1 MHz, in agreement with previously published attenuation coefficients for polyacrylamide gels (Prokop et al. 2003; Takegami et al. 2004).

To confirm that the channels retained a cylindrical shape when the wire was removed, the channels were imaged with a confocal microscope (SP5 MP upright, Leica Microsystems, Germany) (Fig. 1b). CdSe/ZnS core-shell type quantum dots (Sigma-Aldrich), which are large enough not to diffuse into the gel, enabling the channel edge to be clearly delineated, were introduced into the channel to provide fluorescence.

### Ultrasound experiments

The hydrogel (containing the channel) was placed in a tank of de-gassed and de-ionised water under a 40 × water immersion objective lens (model: LUMPLFLN, numerical aperture: 0.8, working distance: 3.3 mm; Olympus, Tokyo, Japan). A focused transducer (model: A303 S-SU, diameter: 13 mm, focal distance: 15.2 mm, centre frequency: 1 MHz; Olympus, Essex, UK) was placed to target the centre of the focal plane of the lens. This alignment was performed using a needle hydrophone (diameter: 0.2 mm, Precision Acoustics, UK). The hydrophone was also used to calibrate the peak-rarefactional pressure *in situ.* An LED light source (KL 2500, Schott, Dorset, UK) provided illumination from below (Fig. 1a).

Each sample was sonicated with a single pulse (pulse length: 10 ms, centre frequency: 1 MHz, peak-rarefactional pressure: 600 kPa). The transducer was driven by sinusoidal pulses generated by a function generator (33500 B Series, Agilent Technologies, Santa Clara, CA, USA) and passed through a 50-dB amplifier (E&I, Rochester, NY, USA). A frequency of 1 MHz was chosen as it is commonly used in clinical systems and many proposed applications. Lower-frequency pulses can also penetrate deeper into the body. For the stiffest gels (*E* = 8.7 kPa), a peak-rarefactional pressure of 1 MPa was used instead, as no deformation could be seen at lower pressures.

### Optical imaging of bubbles

**Fig. 2:**
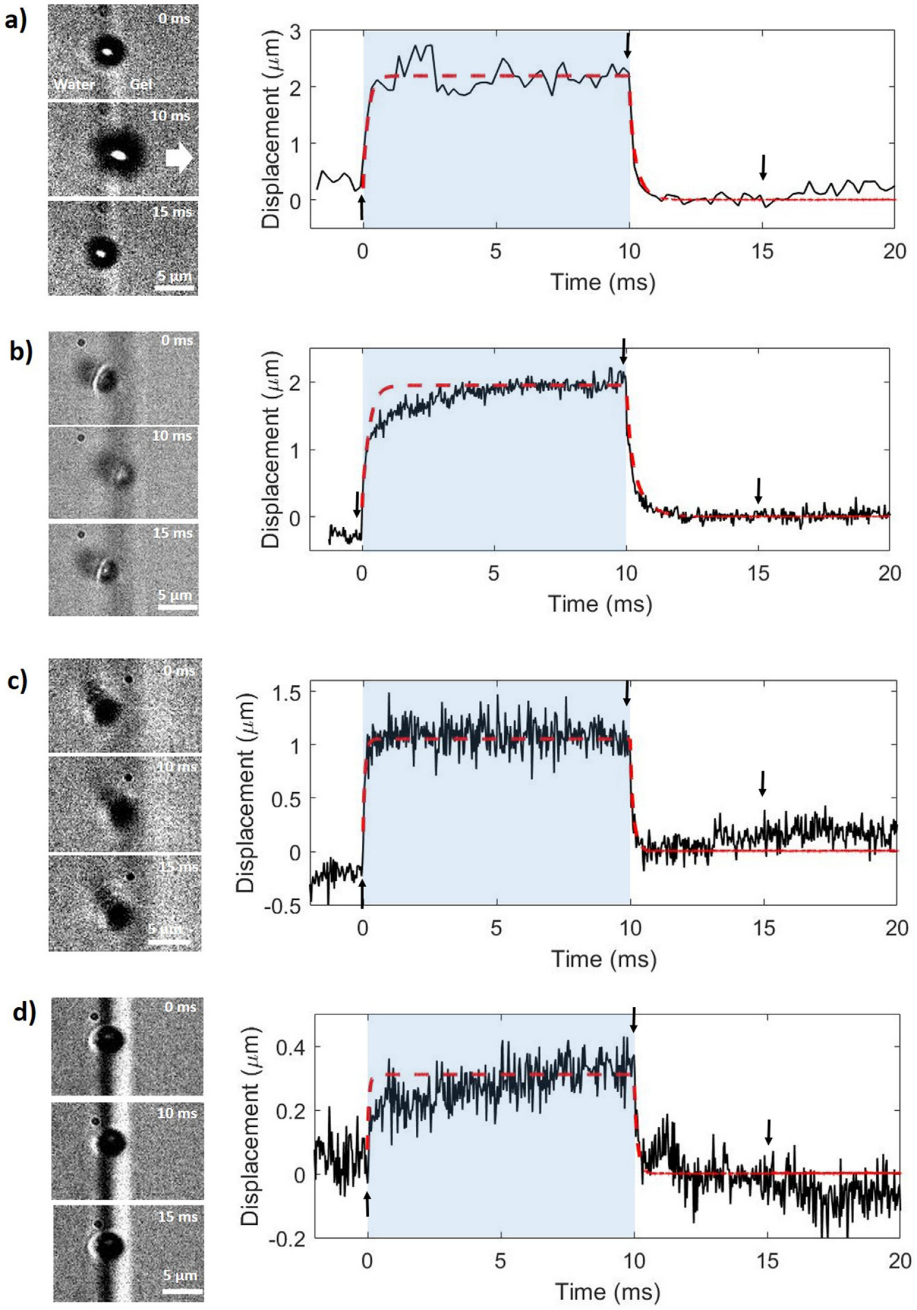
Example indentation curves for four individual bubbles impacting gels with different stiffnesses. Frames from each video are shown on the left, with the *x*-coordinate of the centre of the bubble shown over time in a plot on the right. *Arrows* indicate the points at which each still was taken. The duration of the pulse is shaded in *blue*. Curves fitted to the data based on the mathematical model are represented by *dashed lines in red*. These curves were used to extract the radiation force on each bubble and the viscosity of the gel. The *white arrow* indicates the direction of wave propagation (left–right in all images). (a) Example deformation curve for a 2.0-μm radius bubble indenting a gel with a Young's modulus of 2 kPa. Frame rate: 4,858 frames per second (fps). Parameters extracted from the model are radiation force = 19 nN and viscosity = 0.12 Pa·s. Taken from Supplementary Video S1 (online only). (b) Bubble radius = 1.5 μm. Gel Young's modulus = 2 kPa. Frame rate = 31,197 fps. Radiation force = 13 nN, viscosity = 0.18 Pa·s. Taken from Supplementary Video S2. (c) Bubble radius = 1.5 μm. Gel Young's modulus = 4.5 kPa. Frame rate = 31,197 fps. Radiation force =15 nN, viscosity = 0.12 Pa·s. Taken from Supplementary Video S3 (online only). (d) Bubble radius = 1.6 μm. Gel Young's modulus = 8.7 kPa. Frame rate = 31,197 fps. Force = 4.5 nN, viscosity = 0.2 Pa·s. Taken from Supplementary Video S4 (online only).

**Fig. 3:**
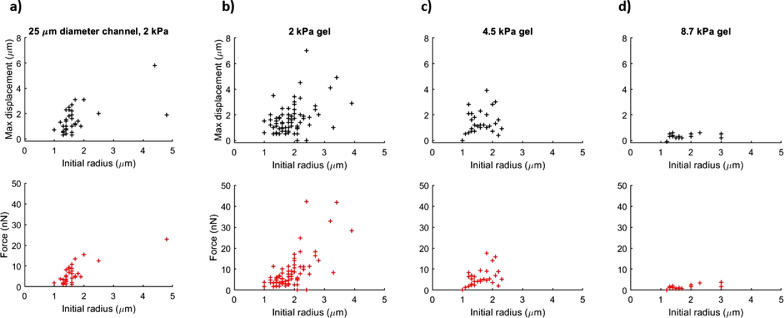
Bubble maximum indentation depth (top row) and radiation force (bottom row) versus bubble radius for three different gel stiffnesses. (a) Maximum indentation depth and force for bubbles in 25-μm-diameter channels in gels with Young's modulus of 2 kPa. (b–d) Maximum indentation depth and force for bubbles in 100-µm-diameter channels in gels with Young's moduli of (b) 2 kPa, exposed to a 600-kPa pulse, (c) 4.5 kPa, exposed to a 600-kPa pulse, and (d) 8.7 kPa, exposed to a 1-MPa pulse. There is no significant difference in values of force or indentation depth between gels with Young's moduli of 2 and 4.7 kPa. Both force and maximum indentation depth were significantly lower in the 8.7-kPa gel channels. There is no significant difference in values of force or indentation depth between the two channel diameters.

Individual microbubbles were optically tracked before, during and after each ultrasound pulse using high-speed microscopy. Videos were obtained using a Chronos 1.4 monochrome high-speed camera (Kron Technologies Inc., Burnaby, BC, Canada), connected to the objective lens *via* a custom optical setup incorporating a tube lens and corner mirror (ThorLabs, Newton, NJ, USA). The setup was placed on an actively damped vibration isolation table (Vision Isostation, Newport, Irvine, CA, USA). The pixel pitch on all videos was 0.16 µm. Most videos were captured at 4,858 fps (Figs. 2a and 3), with a frame size of 304 × 600 pixels, although some images of single bubbles were taken at 31,197 fps (Fig. 2b–d), with a frame size of 336 × 120 pixels, to track the shape of the deformation curve in more detail. The videos and still images shown in the figures were cropped to a size of 304 × 120 pixels to focus on the bubble in the image centre.

### Analysis of raw videos

Bubble movement from the videos was tracked in MATLAB (The MathWorks, Natick, MA, USA). Bubbles were identified in images using an arbitrary intensity threshold technique, which was generally reliable as bubbles had a high contrast compared with the homogeneous background of the channel. Bubbles were isolated from the images using a Hough transform (Duda and Hart 1972). This transform fits a circle to the bubbles, providing a bubble radius and centroid for each frame. Analysis was based on an initial radius of the bubble taken from the first frame of each video.

Although the displacement of the boundary in contact with the bubble will likely be slightly greater than the displacement of the centre of the bubble owing to the bubble's volumetric expansion during the pulse, tracking the centre was deemed to be the most reliable approach to tracking displacement at these frame rates, because of the blurring of the bubble edge due to its radial oscillations.

Background motion of the gel was observed and was owing primarily to environmental vibrations. Because the rapid relaxation of the bubble after the pulse occurred over far shorter timescales than the motion of the background, this background motion did not affect estimates of maximum indentation depth. However, motion correction of the video background was performed using a cross-correlation technique, by tracking the movement of a region of channel wall far from the bubble. This provided an additional control to ensure the motion recorded was of the bubble relative to the channel and did not include any motion of the surroundings. It also enabled the shape of the deformation curves to be assessed more reliably.

Bubbles were excluded from analysis under several conditions: they exhibited no clear response to ultrasound at all; their diameters were <1 µm (as they could not then be reliably identified as spherical bubbles, as opposed to lipid droplets or solid fragments); they did not return back into the channel after the pulse (indicative of gel disruption); they had obvious interactions with neighbouring bubbles or were within 100 μm of another bubble; they were near an obvious imperfection in the gel; or they were significantly out of focus in the initial or final frames.

For each video, the maximum indentation depth was calculated from the relaxation of the bubble after the pulse. The x-coordinate of the videos was used as this is the direction of sound propagation. To reduce the effect of noise, the maximum x-coordinate was taken from the average value over the last 1 ms of the pulse. The final resting state was taken as the average x-coordinate between 2 and 3 ms after the pulse, to ensure the bubble had enough time to stop moving. The maximum indentation depth was defined as the difference between these maximum x-coordinates and the final resting state.

The relaxation of the bubble after the pulse (rather than at the start) was chosen as reference as the bubble can be assumed to be in direct contact with the gel during this. This may not necessarily be the case before the pulse. Because of their small size and consequent low Reynolds number, bubbles will generally not move unless driven directly by either the ultrasound or an elastic force from the gel, over the short timescales of the pulse during which buoyancy can be ignored.

### Modelling

The dynamic response of each bubble was examined using a mathematical model of a bubble indenting into a tissue interface. In this model, the displacement, *u*, of a bubble of radius *R* at a viscoelastic interface (Fig. 1c), exposed to an external force with an amplitude *f*_0_ and duration *τ,* is given by the equation (Koruk and Choi 2018, 2019)

(1) $$u=-\frac{jf_{0}}{6\pi R\left[1-{\left(1-\frac{u}{R}\right)}^{3}\right]}F^{-1}\left[\frac{\left(e^{j\omega \tau }-1\right)\left(3-jkR\right)}{\omega \left(G-j\eta \omega \right)\left(1-jkR-\frac{1}{6}k^{2}R^{2}+\frac{1}{18}jk^{3}R^{3}\right)}\right]$$

where *G, ρ* and *η* are the gel shear modulus, density and viscosity, respectively, $k=\omega /\sqrt{\left(G/\rho )(1-j\omega \eta /G\right)}$ is the wavenumber of the shear wave with frequency *ω* and$\;F^{-1}$ represents the inverse Fourier transform. The shear modulus, *G,* is related to the Young's modulus, *E*, by *G* = *E*/2(1 + *ν*), where *ν* is Poisson's ratio, taken as 0.45 for the gels. Here, the excitation duration *τ* was divided into *N* points (*e.g.,* 1000), and the calculations were repeated over the entire period of interest using MATLAB.

In this model, the gel is modelled as a linear viscoelastic medium and is assumed to be isotropic, homogeneous and incompressible. The model does not account for bubble radial oscillations, which occur in the experiment. The model assumes that the bubble is indenting a plane boundary. In the experiment, the boundary is slightly curved, because the channel is cylindrical. Because the bubble diameter is significantly less than the channel radius of 100 μm, this was assumed to be of minimal significance in modelling.

For every bubble, the time-averaged force was estimated based on the maximum indentation depth, bubble initial radius and gel Young's modulus. For a small sample of bubbles which were imaged at higher frame rates, the viscosity of the gel was also inferred from the shape of the deformation curve including the rate of relaxation after the pulse by minimising the error between the experimental and theoretical dynamic responses of the bubble.

## Results

### Feasibility and qualitative observations

Many instances of a single bubble reversibly deforming a gel boundary were observed. Four different example cases are illustrated in Figure 2, in which the microbubble (1.5–2.0 *μ*m in radius) reversibly deformed a hydrogel (*E* = 2, 4.5 and 8.7 kPa) when exposed to ultrasound. These bubbles produced tissue loading–unloading curves that were similar to those produced by other indentation-based methods (Briscoe et al. 1998; Cheng and Cheng 2005): (i) initial position, (ii) rapid tissue deformation (loading), (iii) a maximum or steady-state deformation (holding), (iv) relaxation of the deformation (unloading) and (v) the final position. The maximum indentation depth of several micrometres was reached rapidly after the start of the pulse. For some bubbles, this was achieved after only tens of microseconds (Fig. 2a, 2c), whereas others had a slightly more gradual increase (Fig. 2b, 2d). In general, we can see a very rapid initial motion, followed by a much slower motion over several milliseconds to the steady-state displacement. The bubbles remained in this position until rapidly relaxing to close to their initial state at the end of the pulse. Microbubbles almost always returned to within the channel after the pulse, typically over 1–2 ms.

Several control videos were obtained of gels without any bubbles, to track the motion of the channel wall caused by the radiation force on the gel alone. No motion could be identified as being caused by the pulse, beyond some slight background vibrations that were present without the ultrasound.

### Radiation force and gel viscosity estimation

The response of each bubble was studied using the mathematical model described previously to infer the radiation force on the bubble and the viscosity of the gel based on experimental observations of the bubble indentation curve.

When exposed to a 600-kPa ultrasound pulse, the force for a 2-*μ*m-radius bubble reaching a maximum displacement of around 2.2 *μ*m in a 2-kPa gel was estimated to be 19 nN (Fig. 2a). The viscosity of the gel was estimated to be 0.12 Pa·s for this experiment. The force decreased to 13 nN for a 1.5-*μ*m-radius bubble, and a maximum displacement of around 1.9 *μ*m was produced (Fig. 2b). The viscosity of the gel for this setup was predicted to be 0.18 Pa·s. The force was estimated to be 15 nN for a 1.5-*μ*m-radius bubble and a maximum displacement of around 1 *μ*m for a 4.5-kPa gel (Fig. 2c), the viscosity being 0.12 Pa·s. The force was 4.5 nN for a 1.6-*μ*m-radius bubble and a maximum displacement of around 0.3 *μ*m for an 8.7-kPa gel, when exposed to 1-MPa-peak-rarefactional-pressure ultrasound pulse (Fig. 2d), where the viscosity is 0.2 Pa·s.

The maximum displacement depends on gel stiffness and bubble radius, as well as the force magnitude. As expected, displacement decreases as gel stiffness increases to 8.7 kPa. It is seen that the force level also decreases with gel stiffness, however, even when the ultrasound pressure was increased. Potential reasons for this, including the assumed material properties, are listed later in the Discussion. Overall, it was seen that the force was typically of the order of 10 nN, and the viscosities of all three gels were estimated at between 0.1 and 0.2 Pa s.

### Quantitative measurements

The maximum displacements into the gel of more than 150 individual bubbles were measured (Fig. 3). The majority of these bubbles were between 1 and 3 µm in radius, because larger bubbles were scarce. Maximum indentation depths for these bubbles were typically less than 4 µm, although this varied significantly, even between bubbles of very similar sizes.

The indentation depths of the bubbles plotted in Figure 3 were compared across the different gel stiffnesses and channel diameters. In general, the maximum displacement decreased as the gel stiffness increased (Fig. 3b–d). The indentation depths of bubbles in the 100-µm channel of 2 kPa at 600 kPa were significantly higher than the indentation depths of bubbles in the 100-µm channel of 8.7 kPa at 1 MPa (one-way multivariant analysis of variance (MANOVA) *p* value: 0.00015) (Fig. 3b, 3d). There was no statistically significant difference between indentation depths of bubbles in the 100-µm channels of 2 and 4.5 kPa (*p* = 0.12) (Fig. 3b, 3c). There was no statistically significant difference between the 25- and 100-µm channels of 2 kPa (*p* = 0.26) (Fig. 3a, 3b).

### Additional qualitative observations

In addition to the bubble indentation results presented above, several behaviours were observed that are important to note. Many of the largest bubbles (radius >5 µm) exhibited surface modes or non-spherical behaviours, which could clearly be seen even at the low frame rates used here. These surface modes may have been present in smaller bubbles, but the spatial resolution made this impossible to verify. The higher resonance frequency of smaller bubbles makes this less likely, however. Bubbles sometimes fragmented during the pulse or dissolved shortly (<1 s) thereafter. Bubble dissolution or fragmentation was most often observed in large bubbles (radius >3 µm) as a result of the 1-MPa pulses used in the stiffest gel. Bubbles sometimes moved rapidly along the channel wall in preference to penetrating it. This was most commonly seen in large bubbles and stiffer gels, and may be due to imperfect alignment of the angle of the transducer.

## Discussion

Single microbubbles have been found to reversibly deform soft gels. This indicates that, under the acoustic parameters tested, which are typical of those used in many therapies, the radiation force on a bubble is likely to generate significant local tissue stresses, and potentially micron-scale displacements, in very soft tissues, such as brain tissue. Thus, radial oscillations are not the only way single microbubbles can exert forces on tissue during therapy. The primary radiation force provides a different mechanical interaction, as its force is unidirectional and sustained.

### Mechanical effects of microbubbles

The forces estimated from the mathematical model were typically of the order of a few tens of nanonewtons in the 2- and 4.5-kPa gels (Fig. 3). For the 8.7-kPa gel, these forces were estimated to be lower, even though the bubbles in the stiffer gel were being exposed to higher acoustic pressures. For the parameters tested in our study, almost all bubbles returned to close to their initial position within the channel after the pulse, and so we assume no permanent structural changes were imparted to the gel, as has previously been reported in agarose at higher pressures (Caskey et al. 2009).

There may be several reasons for the smaller deformation with the 8.7-kPa gel. It could be related to effects on the bubble because of the proximity of the more rigid boundary. When a bubble is in contact with a very soft boundary, the effect of the boundary on its acoustic response is relatively small (Doinikov et al. 2012; Helfield et al. 2014), compared with the very significant damping of oscillations and a reduction in natural frequency when a bubble is in contact with a rigid boundary (Garbin et al. 2007; Overvelde et al. 2011). However, the stiffness whereby bubble oscillations become significantly influenced is unclear. It is also plausible that the effective gel stiffness at our indentation frequencies was much higher than the values measured with AFM.

We noticed variation in the rate at which bubbles reached their maximum displacement. This may be due to variations in gel viscosity and stiffness between different gels or regions of the same gel, as a lower gel elasticity with a higher viscosity causes a slower increase in indentation (Koruk and Choi 2018, 2019). Other possible explanations include motion of the bubble relative to the curvature of the channel wall and change in the bubbles’ gas content or shell structures during the long pulse sonications, meaning the force level may not be constant over time.

No difference was observed between bubbles in the 25- and 100-µm-diameter channels of 2 kPa. This indicates, as has been suggested previously (Qin and Ferrara 2007; Hosseinkhah and Hynynen 2012), that confinement within a very soft, acoustically transparent channel, even one that is very small, does not significantly damp bubble oscillations, as would be predicted in a rigid tube.

Very little deformation was observed in stiffer gels (*E* = 8.7 kPa). Small deformations could only be observed at high mechanical index (≥1). However, many lipid-shelled microbubbles rapidly dissolve or fragment at these pressures, as has been reported previously (Borden et al. 2005; Cox and Thomas 2010, 2013; Kwan and Borden 2010). Enabling microbubble-induced indentation of stiffer tissues may therefore require microbubbles that are more resilient to high acoustic pressures. In stiffer tissues, therefore, such as arteries, direct mechanical effects of lipid-shelled microbubbles caused by radiation force are likely to be confined to close to the vascular wall.

### Limitations

There was significant variation in the amplitudes of deformation between apparently identical bubbles, and it was difficult to observe a clear trend with bubble initial radius. There are many potential reasons for this. The stiffness of each gel measured *via* AFM can vary significantly in different regions (Tse and Engler 2010). Previous studies on acoustic radiation force in a free fluid have also reported similar degrees of variation (Dayton et al. 2002), suggesting intrinsic variation in acoustic response between bubbles of similar size. This could be due to differences in shell structure between bubbles (Borden et al. 2006). The bubbles may also partially dissolve or change shell structures during the long pulse sonications, meaning the force level may not be constant over time. Other reasons may include variable formation of standing waves around the lens; inhomogeneities in the acoustic pressure field caused by scattering between different surfaces near the channel; and imperfect alignment of the angle of the transducer.

The shape of the deformation curves predicted by the mathematical model approximately matched those observed experimentally. However, the values of radiation force and viscosity estimated from the model could not be independently verified, and so these results cannot conclusively establish the quantitative validity of the model. Values for the viscosity of the gel can be estimated with low-frequency oscillatory rheometry or indentation tests. However, the relevance of such values to the micron-scale, high-frequency material properties experienced by the bubble here is unclear.

In the supplementary videos (online only), the edge of the channel at the level of the bubble is not precisely delineated because of its curvature in the vertical plane (Fig. 2). For many bubbles, such as in Figure 2a, it is very clear that the bubble passes beyond the channel boundary and into the gel during the pulse. We therefore assume that the relaxation of all bubbles is due to the rebound of the gel after it is deformed. For very small deformations, however, it is difficult to state conclusively whether the bubble relaxation is due to the elasticity of the gel, to deformation of the bubble itself or to continued fluid streaming, without much higher spatial and temporal resolution than was available here. There was often a small discrepancy in initial and final bubble positions. This may be because the bubble was close to, but not quite in contact with, the wall before the pulse, or because of movement along the curved channel wall in the vertical direction parallel to the imaging plane. It may also be due to slight plastic deformation of the gel. This small discrepancy did not affect the data analysis to compare bubbles and extract parameters, as only the motion of the bubble around the end of the pulse was used, during which the bubble can be assumed to be in contact with the gel.

### Clinical relevance

The ultrasound parameters used here are comparable to those used in therapies such as ultrasound blood–brain barrier permeabilisation. These observations indicate that microbubbles have the potential to induce directional micron-scale displacements of tissues in the vicinity of small blood vessels in soft tissues. We have also found an experimental method to estimate the magnitude of microbubble-induced radiation forces on tissues. This research therefore provides a deeper understanding of the forces microbubbles are exerting when generating therapeutic effects.

It is difficult to state conclusively what biological effects such a local force and strain may have on tissue because of the lack of direct optical observation of acoustic cavitation in capillaries. Tight junction disruption is believed to be part of the mechanism of ultrasound blood–brain barrier opening (Sheikov et al. 2004). Tests carried out on tight junction proteins have found that they unfold when extended by less than 500 nm (Spadaro et al. 2017). Although this was tested only on individual proteins, it is still therefore plausible that tissue deformations of several micrometres could disrupt tight junctional integrity.

The material Young's moduli used here are very relevant to an *in vivo* setting. The precise values of Young's modulus estimated for tissue can vary based on the measurement techniques used. However, our gel Young's moduli of 2–8.7 kPa fit well within the range of those measured for several types of healthy soft tissue. In the porcine brain, Young's moduli of 1.787 ± 0.186 and 1.195 ± 0.157 kPa have been measured using indentation techniques for *ex vivo* white matter and gray matter, respectively (Kaster et al. 2011). By use of ultrasound elastography *in vivo*, a Young's modulus of 4.756 ± 0.271 kPa was measured in rabbit brains (Liu et al. 2018). Bovine healthy liver and muscle Young's moduli were measured from 0.43 to 3.15 kPa depending on the sample and method used (Chen et al. 1996).

This study may have relevance to radiation force targeting in molecular ultrasound imaging, as a way of estimating any potential mechanical effects on tissues. The radiation force pulse parameters under investigation for use in molecular imaging vary widely. However, the very rapid motion of the bubbles into the gel observed in these results indicates that significant displacements do not necessarily require long pulses and could instead be imparted on shorter time scales of tens of microseconds.

Much larger pressures (>>1 MPa) were not tested, and so it is unknown whether reversible deformations could be achieved in stiffer materials. However, bubble fragmentation and dissolution were frequently observed at 1 MPa, making it likely that only a small proportion of the bubble type used here would be able to survive long enough at high pressures to reach a stable maximum equilibrium displacement in the gel.

### Potential applications

This study indicates the potential for the radiation force on a microbubble to probe the mechanical properties of soft materials or *in vivo* tissue at micron-scale spatial resolution. If the radius of a microbubble is known, and its radiation force can be independently calibrated, the elasticity of the medium could be estimated from the bubble's maximum indentation depth. Bubbles rapidly reach a stable maximum depth, which is dependent on tissue stiffness and is maintained over several milliseconds during the pulse. Unlike conventional indentation testing, this method could be performed remotely, without requiring clear access to the surface of the material. It could also be performed at far higher frequencies. The rate of relaxation could be measured with a higher-frame-rate optical or acoustic imaging system, meaning microbubble indentation could also be used to estimate material viscosity *in vitro* or in superficial tissues.

## Conclusions

Sustained, localised and reversible material indentation resulting from the primary acoustic radiation force on single microbubbles has been observed in soft tissue-mimicking materials when exposed to typical therapeutic ultrasound pulses. The indentation of a bubble into a soft material has been used to estimate the force on the bubble and the mechanical properties of the medium. This research provides insight into the nature of the forces that microbubbles may exert on tissues during therapy and the degree of tissue displacement that may be induced by single microbubbles within the microvasculature. Finally, if the primary acoustic radiation force applied by a batch of microbubbles could be made more consistent, then this technique could be used to estimate the mechanical properties of soft materials and *in vivo* tissue.

## Appendix Supplementary materials

Video 1: Indentation of a 2.0 \elsamp #x03BC;m radius bubble into a 2 kPa Young\elsamp #x0027;s modulus gel. Frame rate: 4,858 frames per second (fps). Ultrasound pulse length: 10 ms, Peak Rarefactional Pressure (PRP): 600 kPa. Scale bar: 5 \elsamp #x03BC;m. Time from start of pulse is shown in top left corner. Detailed analysis shown in Figure 2 a. Image, video 1

Video 2 Indentation of a 1.5 \elsamp #x03BC;m radius bubble into a 2 kPa Young\elsamp #x0027;s modulus gel. Pulse length: 10 ms. PRP: 600 kPa. Scale bar: 5 \elsamp #x03BC;m. Frame rate: 31,197 fps. Time from start of pulse is shown in top left corner. Detailed analysis shown in Figure 2 b. Image, video 2

Video 3 Indentation of a 1.5 \elsamp #x03BC;m radius bubble into a 4.5 kPa Young\elsamp #x0027;s modulus gel. Pulse length: 10 ms. PRP: 600 kPa Scale bar: 5 \elsamp #x03BC;m. Frame rate: 31,197 fps. Time from start of pulse is shown in top left corner. Detailed analysis shown in Figure 2 c. Image, video 3

Video 4 Indentation of a 1.6 \elsamp #x03BC;m radius bubble into an 8.7 kPa Young\elsamp #x0027;s modulus gel. Pulse length: 10 ms. PRP: 1 MPa. Scale bar: 5 \elsamp #x03BC;m. Frame rate: 31,197 fps. Time from start of pulse is shown in top left corner. Detailed analysis shown in Figure 2 d. Image, video 4
